# Solving the standing-start paradox in track cycling

**DOI:** 10.1098/rsos.252273

**Published:** 2026-09-09

**Authors:** Simon Giraud, Iris Sachet, Emmanuel Brunet, Christophe Clanet

**Affiliations:** ^1^ LadHyX, CNRS - École Polytechnique, Institut Polytechnique de Paris, Palaiseau, 91120 France; ^2^ Fédération Française de Cyclisme, Montigny-le-Bretoneux, France

**Keywords:** track cycling, standing start, performance optimization, sports biomechanics, inertial propulsion

## Abstract

Standing starts in track cycling appear paradoxical: a departure ‘from rest’ can exhibit a non-zero bike velocity at the official signal. We resolve this apparent contradiction by identifying a brief transitional regime, initiated around the gate-release window and preceding steady pedalling, in which coordinated whole-body motion provides the initial impulse. Using direct characterization of the starting gate, high-speed videography of elite specialist starters, anthropometric centre-of-mass (CoM) reconstruction and instrumented crank-torque measurements, we separate three concurrent contributions to the early motion: gate braking, inertial forcing from rapid CoM motion and pedalling propulsion. The resulting dynamical model reproduces the measured velocity–time profiles and explains lawful non-zero start velocities without prescribing them as initial conditions. Optimization analyses show that start performance is governed primarily by the timing of initiation relative to the release window and by the explosiveness of the CoM motion, whereas gearing and mass have only minor effects over this early interval. The model therefore provides practical guidance for improving the first instants of the track-cycling standing start within the rules.

## 1. Introduction

Across sports, the standing start is a decisive determinant of performance. In athletics sprints, power output and onset timing are central [1,2]; in swimming, start efficiency becomes increasingly important as race distance decreases [3]. Despite differences in apparatus and time scale, the common mechanical challenge is to generate a large propulsive impulse from rest under rules that penalize premature motion.

In track cycling, held standing starts are used in the 1 km time trial, the team sprint and the individual and team pursuits; comparable procedures apply in para-cycling timed events [4]. In all these events, the rear wheel is immobilized in a starting gate (Swiss Timing, Corgémont, Switzerland) that opens when an audiovisual countdown reaches zero (figure 1a), thus defining t=0. The practical ‘false-start’ constraint is simple: the front wheel must not cross the start line before the official signal, and the start judge decides whether a start must be stopped for a false start. The perfectly predictable five-second cadence renders the start a distinctive motor task: the athlete must choreograph whole-body motion so that bicycle velocity peaks as the gate unlocks (figure 1b). The quality of this initial launch is therefore important, and has been shown to correlate with early split times in elite team sprint [5].

**Figure 1.**
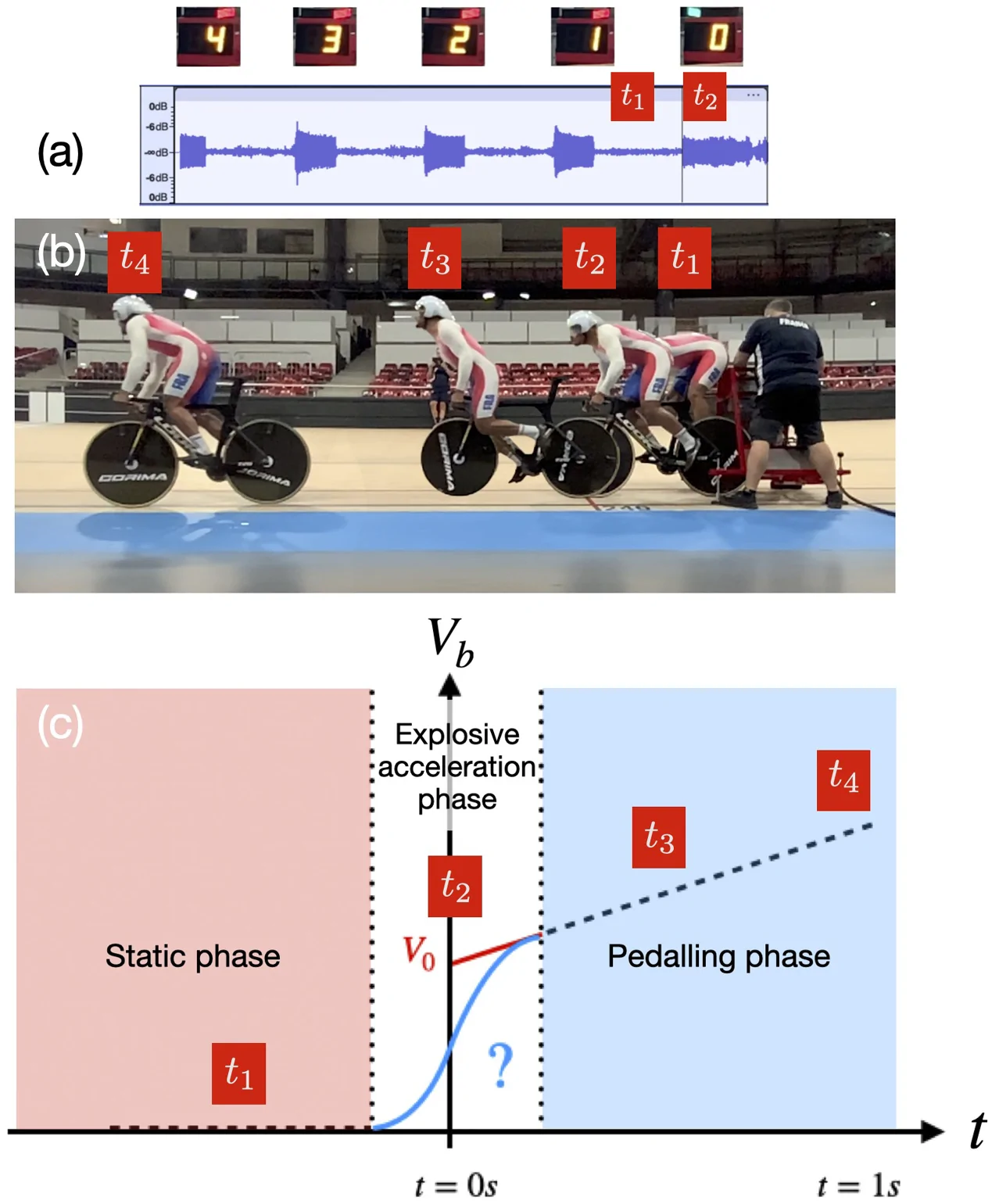
Standing start in track cycling. (a) Visual countdown (−4 s to 0 s) and corresponding sound-pressure amplitude. The gate opens at the vertical black line, marking the official start t=0 visualized by the red light turning to green. (b) Chronophotography of Starter 1 at $t_{1}=-0.5\text{ s}$, $t_{2}=0\text{ s}$, $t_{3}=0.5\text{ s}$ and $t_{4}=1\text{ s}$, showing the progression from gate-constrained posture to forward propulsion. (c) Bike velocity–time trace $V_{b}(t)$: conventional predictive profile with imposed non-zero initial velocity $V_{b}(t=0)=V_{0}$ (red) versus the observed profile (blue), which exhibits a short explosive transitional phase before steady pedalling.

**The standing-start paradox.** Mechanically, the held start in track cycling is often reduced to two phases: a static gate-constrained phase followed by sustained pedalling [6,7]. Yet elite starting riders can present a non-zero bicycle velocity, $V_{b}(t=0)>0$, at the official signal [8]. Figure 1 illustrates this apparent contradiction. The chronophotography in panel (b) shows the progression from the held posture to forward propulsion, while panel (c) compares a conventional profile (in red), in which a non-zero initial velocity is imposed, with an actual trace (in blue). This trace reveals a short explosive transitional regime preceding steady pedalling. This regime, rather than an ad hoc initial condition, is the focus of the present study.

**Scope and structure.** We develop a physical framework that separates and quantifies three contributions to the initial motion of a held standing start: (i) braking by the gate, (ii) inertial forcing from whole-body motion and (iii) pedalling propulsion. In §2, we extract early velocity signatures from high-speed video. In §3, we measure the forces applied to the bicycle during the standing start. The full model is then validated in §4. Finally, in §5, we use the model to identify lawful performance gains in initiation timing and early body kinematics, with practical implications for training and competition.

## 2. Kinematics of standing starts

### 2.1. Material and methods

We analysed the standing-start kinematics of three elite French track cyclists who all serve as starters in the national team sprint programme (two men, Starter 1 and Starter 2; one woman, Starter 3). As specialist starters, these athletes routinely train and compete under the stringent constraints of a held start, which makes them particularly well suited to the present investigation.

Bicycle motion was quantified from high-resolution video recorded at 120 frames s${}^{-1}$. For each trial, we tracked, frame by frame, the front-hub position in the track-fixed Cartesian frame $\left(X_{a},Y_{a}\right)$, whose origin lay on the start line and whose $X_{a}$-axis points along the direction of motion (figure 2). Within this reference frame, the bicycle trajectory was represented by the front-hub coordinates $\left(X_{b},Y_{b}\right)$.

**Figure 2.**
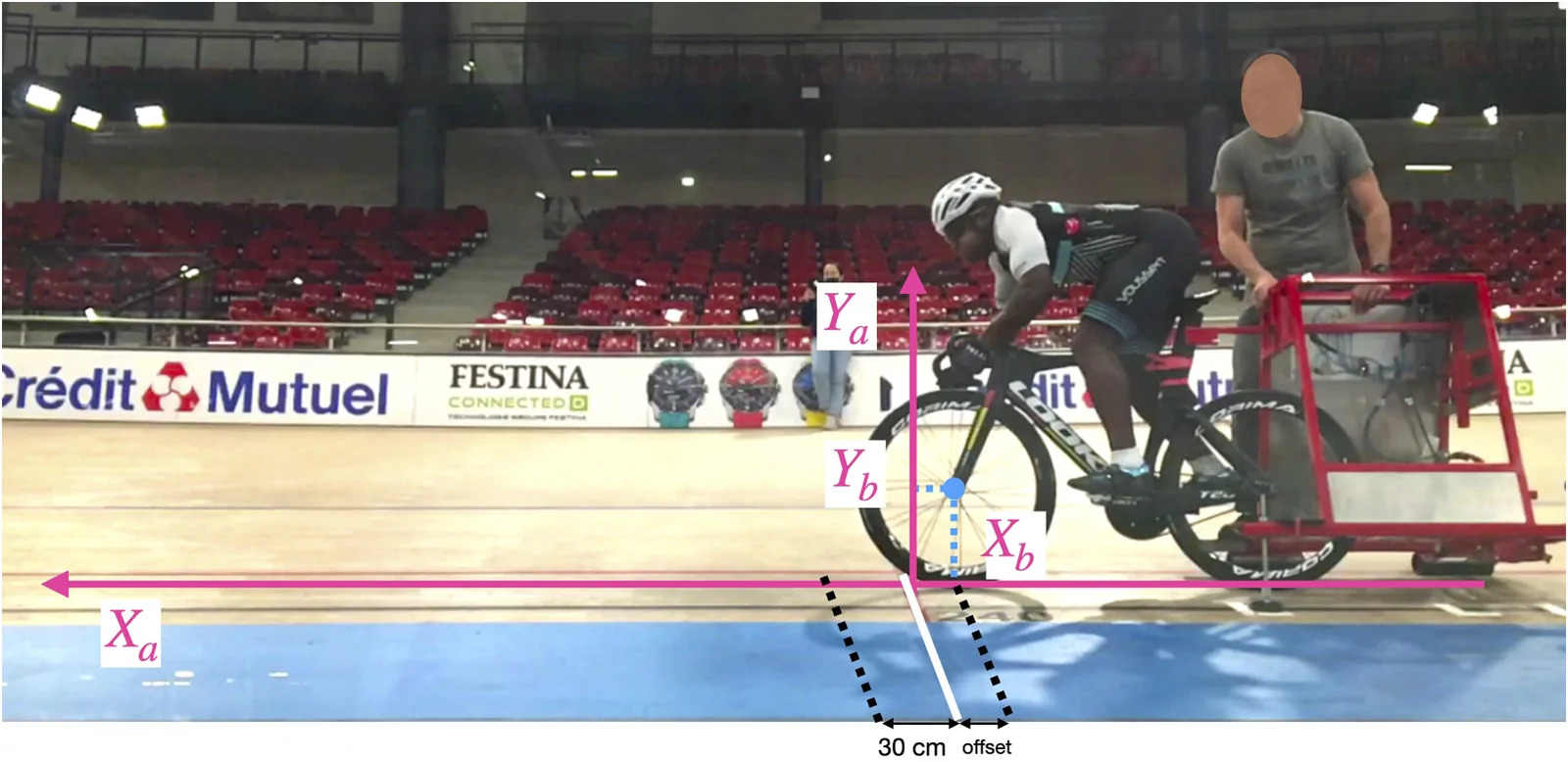
The track-fixed frame $\left(X_{a},Y_{a}\right)$ originates at the start line (white line) and is used to describe bicycle front-hub motion $\left(X_{b},Y_{b}\right)$. $X_{b}(t=0)$ defines the offset.

The offset illustrated in figure 2 is defined by $X_{b}(t=0)$. It is set by the position of the starting gate chosen by the officials and by bicycle frame geometry. For a given starting-gate position, smaller riders generally use smaller frames, which tend to place the front wheel farther behind the start line and therefore increase the offset. Conversely, larger frames tend to reduce the offset.

The longitudinal velocity $V_{b}={\dot{X}}_{b}$ was obtained by differentiating the position time series. The sampling rate provides a temporal resolution of 8.3 ms and, together with spatial calibration, affords sub-centimetre precision; these resolutions are sufficient to resolve the rapid transients that characterize the first instants of motion.

Videos were captured with a fixed camera, always located at the same position, orthogonal to the start line, and using a short exposure time to limit motion blur. Raw position signals obtained from the frame-by-frame analysis were low-pass filtered with a zero-phase Butterworth filter (second order, 12 Hz cut-off). Velocity was computed from the filtered positions using a second-order central finite difference and was then low-pass filtered with a zero-phase Butterworth filter (fourth order, 8 Hz cut-off). The same filtering and finite-difference procedure was used for the centre-of-mass (CoM) trajectories before calculating relative CoM velocities and accelerations.

### 2.2. Velocity profiles: common patterns and differences

The bicycle velocity histories (figure 3) exhibit a common two-phase form, as sketched in figure 1c. Immediately after the signal (t=0), all riders display a brief, sharp surge in acceleration, followed by a phase that can be approximated by near-constant acceleration. Although the general pattern is shared, the timing and magnitude of the early surge vary between athletes: Starter 1 initiates acceleration earlier and attains a higher velocity sooner after t=0. Starter 2 shows a delayed onset relative to Starter 1 but ultimately approaches a similar final velocity. Starter 3 exhibits both a later onset and a lower terminal velocity than the other two riders.

**Figure 3.**
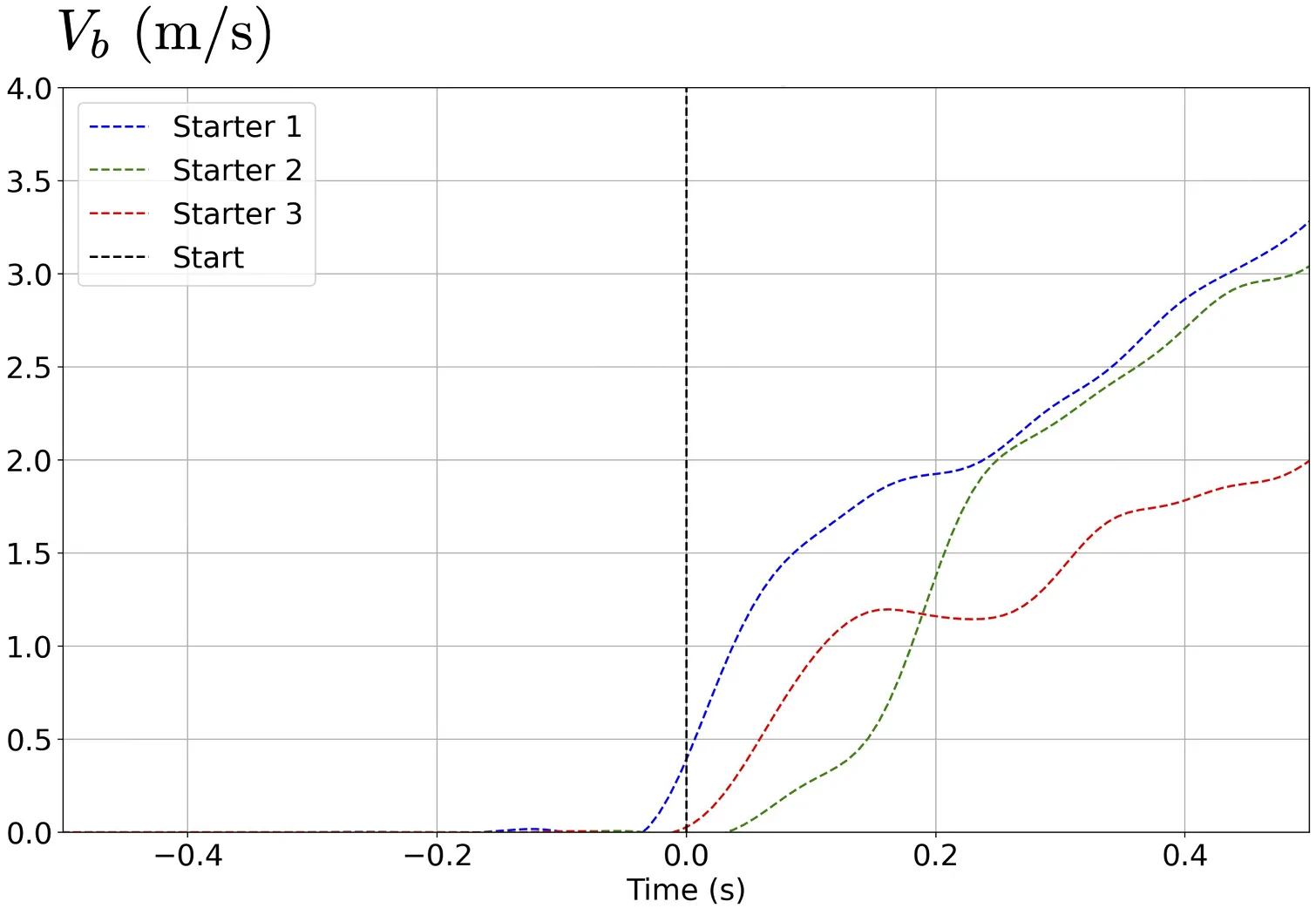
Bicycle velocity profiles for the three cyclists: Starter 1 (blue), Starter 2 (green) and Starter 3 (red). All riders display a two-phase acceleration; athlete-specific differences arise primarily in the timing and magnitude of the initial surge.

Since the area under the velocity curve represents the distance travelled, this early velocity advantage for Starter 1 implies that a non-zero distance is covered before the signal. If the bicycle were positioned exactly on the start line, such motion would potentially result in a false start. A lawful non-zero velocity $V_{b}(t=0)$ is therefore necessarily associated with a finite offset $X_{b}(t=0)$.

### 2.3. $V_{start}$ , $T_{30\;\text{cm}}$ and offset

Table 1 lists a summary of the early-distance performance metrics. Starter 1, who began his gross body motion earliest and was 6 cm behind the line, was the only rider with a non-zero start velocity ($V_{start}=0.4$ m s${}^{-1}$) and reached 30 cm fastest among the three starters (0.23 s). Starters 2 and 3 had larger offsets but began from rest at the signal, showing that offset alone is not sufficient: the phasing of the CoM motion relative to the gate release is also decisive. For comparison, international benchmarks from the 2022 World Championships in Saint-Quentin-en-Yvelines show $T_{30\;\text{cm}}$ values of 0.14 s (GBR) and 0.11 s (AUS), associated with higher initial velocities of 1.2–2.0 m s${}^{-1}$ [8].

**Table 1.** Early-distance performance metrics.

cyclist	offset (cm)	$V_{start}$ (m s${}^{-1}$)	$T_{30\;\text{cm}}$ (s)
Starter 1	6	0.4	0.23
Starter 2	14	0.0	0.35
Starter 3	14	0.0	0.40
GBR 2022	6	1.2	0.14
AUS 2022	4	2.0	0.11

For a rider with $V_{start}>0$, some distance has already been covered before the official signal. This is legal only if the wheel remains behind the start line at t=0. The offset in table 1 is therefore a geometrical allowance: it is the initial distance between the wheel reference point and the start line. It depends on bicycle geometry and gate placement, not on an arbitrary choice made during the start. This explains why a non-zero $V_{start}$ can be lawful, but it also shows why the same offset can lead to different outcomes if the initiation timing differs.

At international level, the last two rows of table 1 show that the initial velocity measured for the best starters, who also achieved the strongest opening 250 m performances, can reach values as high as 2 m s${}^{-1}$.

## 3. Forces applied to the bike during standing start

The dynamics follow directly from Newton's second law applied to the bicycle–cyclist system in the ground frame, using the notation and sign conventions shown in figure 4. Writing the time derivative of total momentum gives

(3.1) $$M_{b}\;{\dot{V}}_{b}+M_{c}\;{\dot{V}}_{c}\;=\;F_{p}-F_{b},$$

where overdots denote time derivatives. The cyclist's ground-frame position decomposes as $X_{c}=X_{b}+(X_{c}-X_{b})$, so that

$$V_{c}\;=\;V_{b}+V_{cb},\;{\dot{V}}_{c}\;=\;{\dot{V}}_{b}+{\dot{V}}_{cb},$$

with $V_{cb}$ the cyclist's velocity relative to the bicycle (zero only if posture is rigidly fixed).

**Figure 4.**
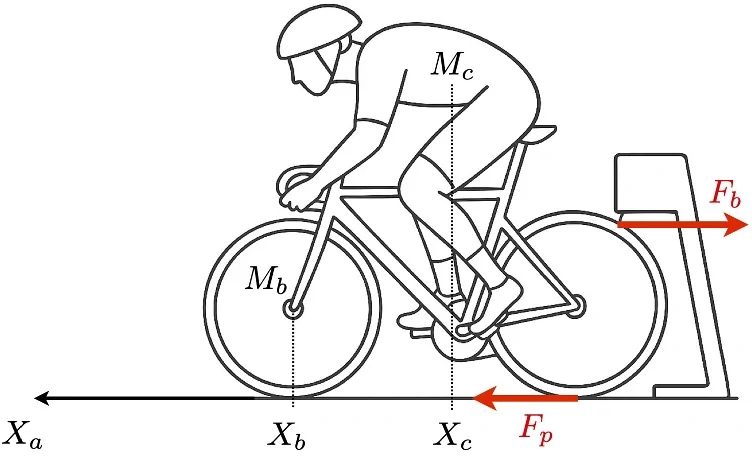
Scheme and conventions. The ground-fixed $X_{a}$-axis is aligned with the direction of travel. Here, $M_{b}$ and $M_{c}$ denote the masses of the bicycle and cyclist, respectively; $V_{b}$ and $V_{c}$ are their ground-frame velocities. The gate exerts a braking force $F_{b}$ on the rear wheel, and pedalling generates a propulsive force $F_{p}$ at the tyre–track contact.

Substituting into equation (3.1) and collecting terms yields

(3.2) $$M\;{\dot{V}}_{b}\;=\;F_{p}-F_{b}\;-\;M_{c}\;{\dot{V}}_{cb},\;M\equiv M_{b}+M_{c}.$$

It is convenient to define the body-induced inertial contribution

(3.3) $$F_{i}\;\equiv \;-M_{c}\;{\dot{V}}_{cb},$$

so that the longitudinal balance takes the compact form

(3.4) $$M\;{\dot{V}}_{b}\;=\;F_{p}-F_{b}+F_{i}.$$

Equation (3.4) makes explicit the three active contributions that govern forward motion: propulsive drive from pedalling ($F_{p}$), the transient gate resistance ($F_{b}$) and an inertial term ($F_{i}$) arising from whole-body motion relative to the bicycle.

The starting gate has two distinct mechanical actions. First, it acts as a one-sided geometrical constraint: while the bicycle is held in the gate, the rear wheel cannot move backwards. Second, it exerts a finite braking force on the rear wheel, denoted $F_{b}(t)$, which opposes forward motion and decreases during release. The bicycle can therefore only move forwards from the held position, and forward motion starts only when the net driving force becomes positive.

To express this constraint, we define the net forward drive:

$$D(t)=F_{p}(t)+F_{i}(t)-F_{b}(t).$$

While the bicycle is still blocked in the gate, a unilateral reaction $F_{r}$ prevents rearward motion:

(3.5) $$M\;{\dot{V}}_{b}=D(t)+F_{r},\;F_{r}=\left\{ \begin{array}{clclc} & -D(t), & \; & \text{if }X_{b}=X_{b,\text{start}}\text{ and }D(t)<0, & \\ & 0, & \; & \text{otherwise.} & \end{array}\right.$$

Thus, when D(t)<0, the reaction of the gate balances the backward tendency and the bicycle remains at rest. When D(t)>0, the reaction vanishes and the bicycle accelerates forwards under the measured contributions $F_{p}$, $F_{i}$ and $F_{b}$.

Aerodynamic drag and rolling resistance are neglected over the first 30 cm because the velocities are small and the time interval is very short. The rotational inertia of the wheels is also negligible at the present level of accuracy. If it were included, rolling without slip would add an effective mass for two wheels [9]:

$$M_{\text{rot}}=\frac{2I_{w}}{R^{2}}.$$

For a representative track wheel of mass $M_{\text{wheel}}≃1\;\text{kg}$, the inertia-equivalent mass is of order $\frac{I_{w}}{R^{2}}≃0.5\;M_{\text{wheel}}$, so that the two wheels together contribute only $M_{\text{rot}}≃1\;\text{kg}$ [10]. This is approximately 1% of the bicycle–rider mass and is small compared with the uncertainty associated with the measured early inertial impulse. We therefore retain $M=M_{b}+M_{c}$ in the model. The next subsections quantify the dominant contributions and we use them to build and validate the full dynamical model.

### 3.1. The braking force $F_{b}$

#### 3.1.1. Experimental findings

Figure 5 shows the Union Cycliste Internationale (UCI) Swiss Timing Starting Gate (panel a) and the braking force $F_{b}(t)$ it exerts on the rear wheel (panel b). These measurements are detailed in appendix A to establish the magnitude and timing of the constraint imposed just before motion onset. It is first shown that the normal clamping load increases approximately linearly with regulator pressure (slope ≈43.4 N bar${}^{-1}$); at the competition standard 6 bar setting we measured $F_{n}\approx 278$ N. Slip-release tests yielded a mean effective friction coefficient of μ≈1.33, implying a pre-release braking force $F_{b,\max}\approx \mu F_{n}\approx 370$ N. Second, time-resolved traces show that the gate begins to open immediately after the official signal and completes release within a brief window of ΔT≈0.06 s, during which the braking force decays smoothly from its plateau to near zero. In practice, the residual action of the gate vanishes within the first few centimetres of travel.

**Figure 5.**
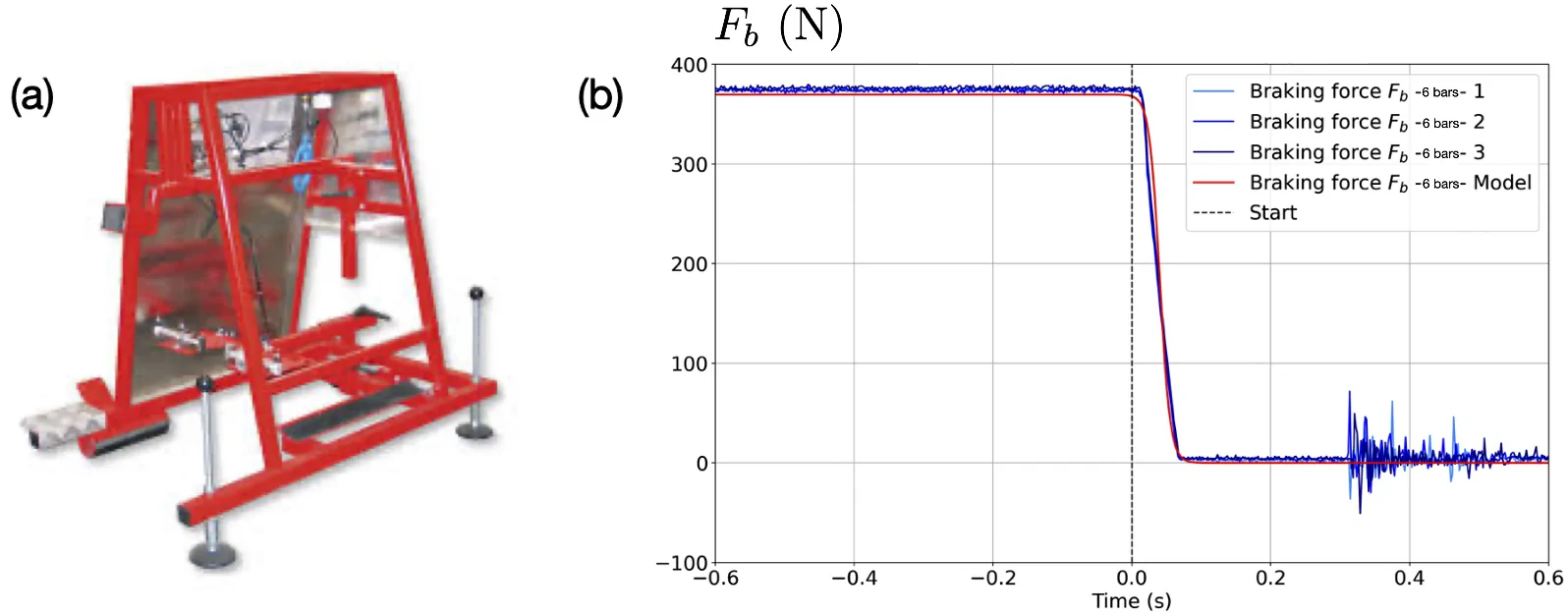
Starting-gate characterization: (a) the Swiss Timing Starting Gate used in UCI World Cup, World Championships and Olympic Games (b) comparison between modelled braking force (equation (3.6)) and experimental measurements at 6 bar.

#### 3.1.2. Model for $F_{b}(t)$

To model the time dependence of the braking force $F_{b}(t)$ presented in blue in figure 5b, we use the function:

(3.6) $$F_{b}(t)\;=\;\frac{F_{b,max}}{2}\left[1-\tanh\;\left(\frac{t-(T_{o}+\Delta T/2)}{\Delta T/2}\right)\right],$$

with $F_{b,\max}=370$ N, $T_{o}$ the onset of gate motion (in figure 5b, $T_{o}=0$) and ΔT=0.06 s the opening duration. This hyperbolic-tangent profile reproduces the measured plateau-and-decay behaviour without introducing discontinuities, and the red fitted curve in figure 5b closely captures the experimental force trace.

### 3.2. The inertial force $F_{i}$

The inertial contribution arises from the rider's deliberate motion relative to the bicycle during the first instants of the start. In particular, the rider moves the whole-body CoM rearwards and then forwards relative to the bicycle. When this motion is appropriately timed with the release of the starting gate, it produces a short but powerful inertial action on the bicycle.

#### 3.2.1. Methods

High-definition video (120 Hz) was recorded with a fixed camera positioned orthogonally to the start line. For each trial, the joint centres used in the segment model were digitized frame by frame. Their coordinates are denoted $\left(X_{i},Y_{i}\right)$, with i=1,…,8, and are expressed in a bicycle-fixed coordinate frame (X,Y) presented in figure 6. The origin of this frame is located at the handlebar, so that the hands have coordinates (0,0). The X-axis points towards the rear wheel and the Y-axis points upwards. This convention gives positive coordinates for the whole-body CoM $\left(X_{G},Y_{G}\right)$, but it also means that the X-axis is opposite to the forward direction of motion so that $V_{cb}=-{\dot{X}}_{G}$.

**Figure 6.**
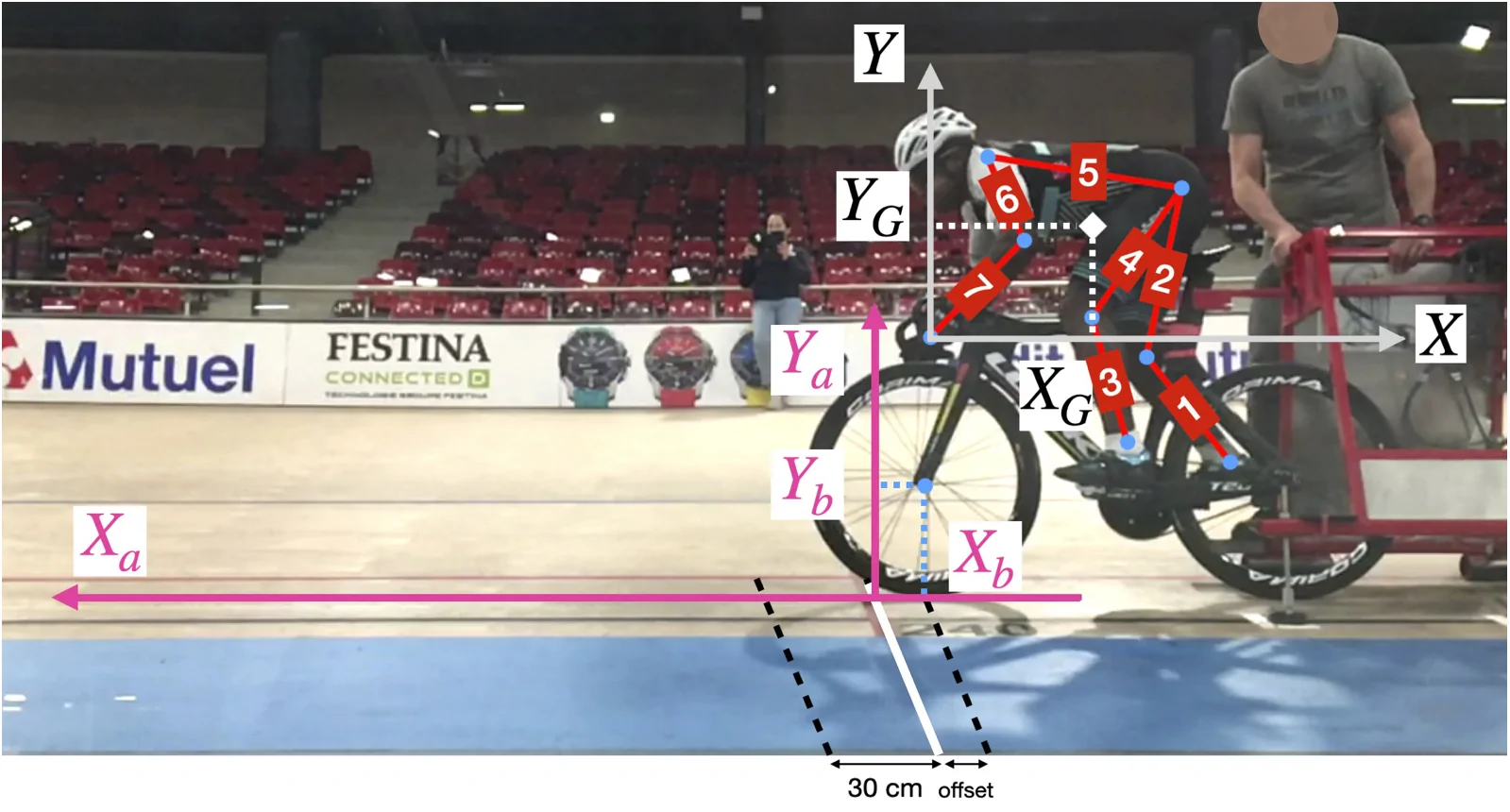
Example of joint and CoM tracking during the start. Blue dots: detected joint centres; white diamond: whole-body CoM; red segments: body parts used for CoM estimation. The local coordinate frame used for CoM reconstruction has its origin at the handlebar, with X pointing towards the rear wheel and Y pointing upwards.

From these marker coordinates, we computed the segmental and whole-body CoM using sex-specific mass fractions and longitudinal CoM positions from de Leva [11]. The same zero-phase filtering and finite-difference procedure as for the front-hub trajectory was then applied to the CoM coordinates. The step-by-step reconstruction procedure is detailed in appendix B.

#### 3.2.2. Results

Across riders, the CoM follows a characteristic rearward-to-forward path relative to the bicycle, consistent with deliberate loading followed by a projection of the body mass. Figure 7a–c shows representative CoM trajectories from B to E for Starters 1–3, with markers every 0.05 s and the start signal indicated by diamonds; panel (d) reports the corresponding horizontal CoM velocity $V_{cb}=-{\dot{X}}_{G}$ relative to the bicycle in the direction of motion ($V_{cb}>0$ means that the CoM moves towards the front wheel).

**Figure 7.**
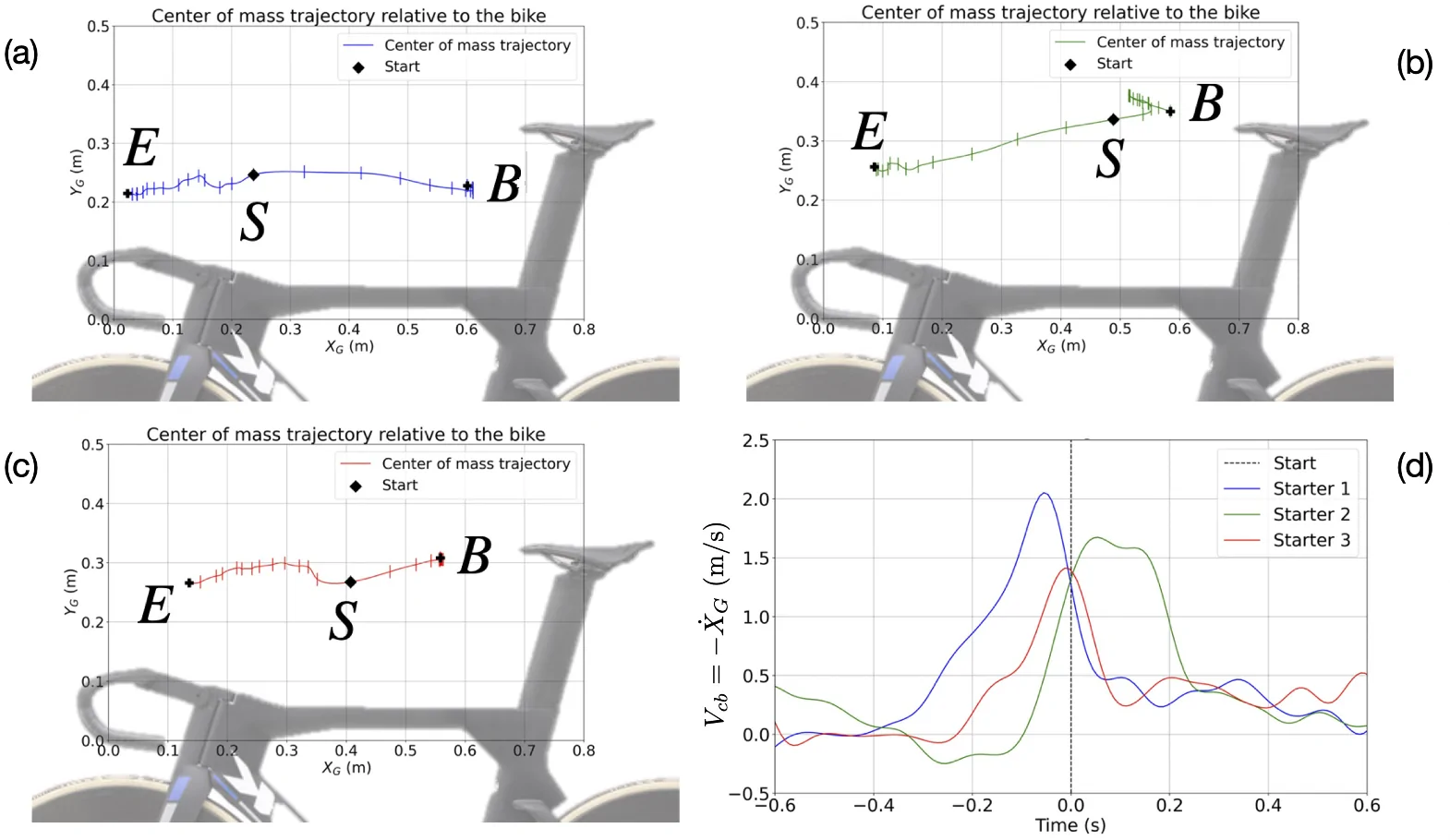
Start-dynamics analysis. (a–c) CoM trajectories relative to the bicycle for Starters 1–3; diamonds denote the start signal; markers are spaced by 0.05 s; B/E indicates the beginning/end of the gross body motion. In each graph, the diamond stands for t=0. (d) Horizontal CoM velocity relative to the bicycle in the forward direction: $V_{cb}=-{\dot{X}}_{G}$.

Starter 1 exhibits the most pronounced rearward displacement before the signal and achieves the highest peak forward CoM velocity, approximately 2.0 m s${}^{-1}$. Starter 2 follows a slightly more descending path, consistent with a different weight-transfer strategy, and peaks near 1.6 m s${}^{-1}$. Starter 3 is intermediate, with a peak near 1.4 m s${}^{-1}$. In all cases, the peak forward CoM velocity is followed by a rapid deceleration of the CoM relative to the bicycle. It is this deceleration, when phased with gate release, that produces the largest positive inertial force on the bicycle. The peak velocity of the CoM is therefore not sufficient by itself; the relevant dynamical quantity is the relative acceleration of the CoM, together with its timing.

#### 3.2.3. Evaluation of $F_{i}(t)$

The inertial force defined in equation (3.3) is $F_{i}(t)=-M_{c}\;{\dot{V}}_{cb}(t)$. Since $V_{cb}=-{\dot{X}}_{G}$ it takes the form,

(3.7) $$F_{i}(t)=-M_{c}\;{\dot{V}}_{cb}(t)=M_{c}\;{\ddot{X}}_{G}(t).$$

A propulsive inertial force ($F_{i}>0$) occurs when ${\ddot{X}}_{G}>0$, that is, when the CoM is decelerating after its forward motion towards the frontwheel.

Figure 8 reports $F_{i}(t)$ for all three riders. Peak values arise within the first 0.2 s after the signal, reflecting the brevity of the transitional regime identified in the kinematic analysis.

**Figure 8.**
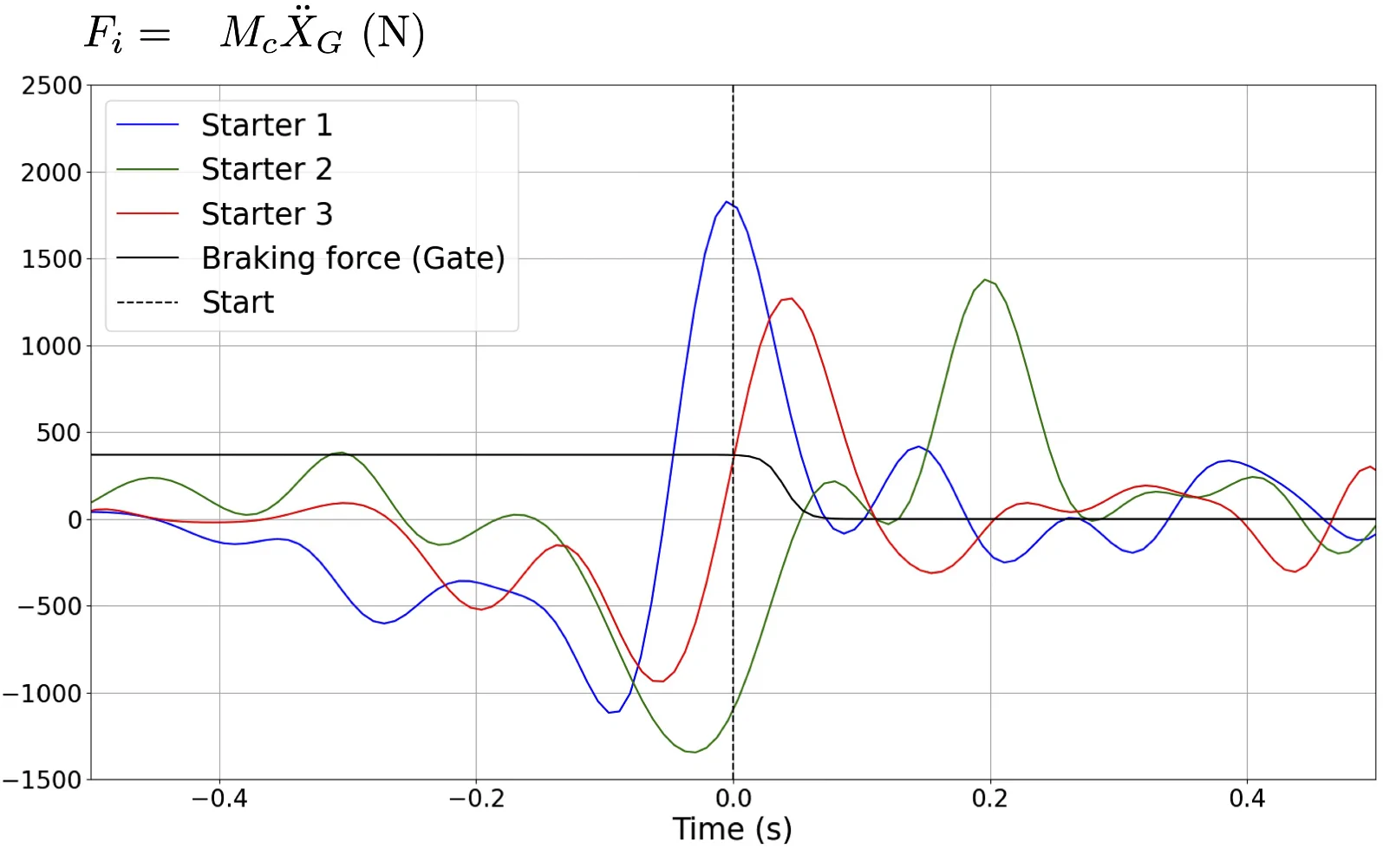
Time evolution of the inertial force $F_{i}=M_{c}{\ddot{X}}_{G}$ for Starter 1 (blue), Starter 2 (green) and Starter 3 (red). The vertical axis is force in newtons; peaks occur within the first 0.2 s, indicating the brevity of the transitional regime and athlete-specific differences in timing and magnitude.

#### 3.2.4. Interpretation and role

The inertial term plays a decisive role at the instant of release. For Starter 1, $F_{i}$ reaches about 1800 N (figure 8), substantially exceeding the peak gate resistance (approx. 370 N) and thereby enabling a rapid transition out of the static phase. In the longitudinal balance, this body-driven impulse provides the initial forward drive before sustained pedalling becomes dominant. The magnitude and timing of ${\ddot{X}}_{G}$ therefore explain the observed differences in onset timing, early acceleration, and the time to short benchmarks such as 30 cm. They also frame the optimization questions addressed later in the paper.

### 3.3. Pedalling force $F_{p}$

During a standing start the rider generates a propulsive pedalling force $F_{p}$ through the drivetrain while coordinating whole-body motion. Pedalling dynamics were recorded with an instrumented crankset sampling at 200 Hz, from which we obtained the instantaneous crank torque $\Gamma_{p}(t)$ and its timing relative to the start signal (t=0).

#### 3.3.1. Force transmission

Figure 9a summarizes how pedal effort is converted into forward drive. Let $F_{nl}$ and $F_{nr}$ denote the pedal forces perpendicular to the left- and right-hand crank arms, and let ℓ be the crank length. The net crank torque is then

(3.8) $$\Gamma_{p}(t)\;=\;(F_{nl}+F_{nr})\;\ell .$$

Assuming negligible drivetrain losses and a gear ratio $G=R_{1}/R_{2}$, the torque at the rear wheel is $\Gamma_{p}/G$. The corresponding propulsive force at the tyre–track contact is therefore

(3.9) $$F_{p}(t)\;=\;\frac{\Gamma_{p}(t)}{R}\;\frac{1}{G}\;=\;\frac{(F_{nl}+F_{nr})\;\ell }{R}\;\frac{1}{G},$$

where R is the effective rear-wheel radius. This relation makes explicit how crank leverage and gearing scale pedal forces into forward thrust.

**Figure 9.**
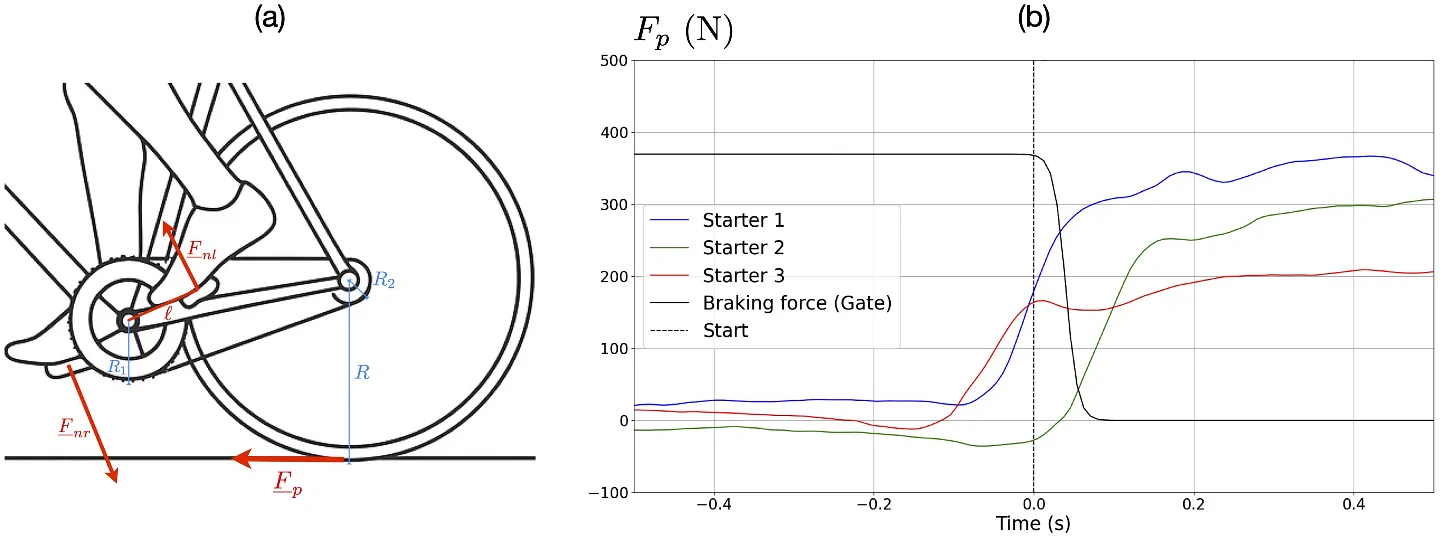
(a) Schematic of pedalling-force transmission from crankset to rear wheel. (b) Time evolution of pedalling force $F_{p}$ for Starter 1 (blue), Starter 2 (green) and Starter 3 (red).

#### 3.3.2. Results

The gear ratios used were G=48/13 for Starter 1, G=54/14 for Starter 2 and G=54/11 for Starter 3. The resulting $F_{p}(t)$ traces are shown in figure 9b. Once the gate's braking action has decayed, $F_{p}$ sustains bicycle acceleration; peak propulsive forces are of the order of a few hundred Newtons. Inter-athlete differences in both onset and rate of increase of $F_{p}$ are observed. In the compact balance $M\;{\dot{V}}_{b}=F_{p}-F_{b}+F_{i}$, pedalling becomes the dominant term immediately after the short transitional regime in which the inertial contribution $F_{i}$ and the gate braking $F_{b}$ coexist. The quasi-plateau observed in figure 9b for $F_{p}$ at t>0.2 s therefore suggests a near-constant acceleration.

## 4. Model validation

### 4.1. The full model

Having quantified the three principal contributions—pedalling propulsion $F_{p}$, the body-induced inertial term $F_{i}$ and the gate capacity $F_{b}$—we combine measurements and constitutive relations into a single dynamical description of the start. Following equation (3.4), the longitudinal acceleration of the bicycle obeys

(4.1) $$M\frac{dV_{b}}{dt}=\frac{\Gamma_{p}(t)}{R}\;\frac{1}{G}+M_{c}\;{\ddot{X}}_{G}(t)-F_{b}(t)+F_{r}(t),$$

with $F_{b}(t)$ given by equation (3.6) and $F_{r}(t)$ by equation (3.5). Motion starts when the right-hand side becomes positive.

### 4.2. Validation

The comparison between model-predicted velocities and video-measured velocities for the three starters is shown in figure 10. The model reproduces the observed velocity–time histories with good fidelity: predictions are accurate for Starters 1 and 3, while for Starter 2 a modest delay in predicted onset yields lower early velocities than measured.

**Figure 10.**
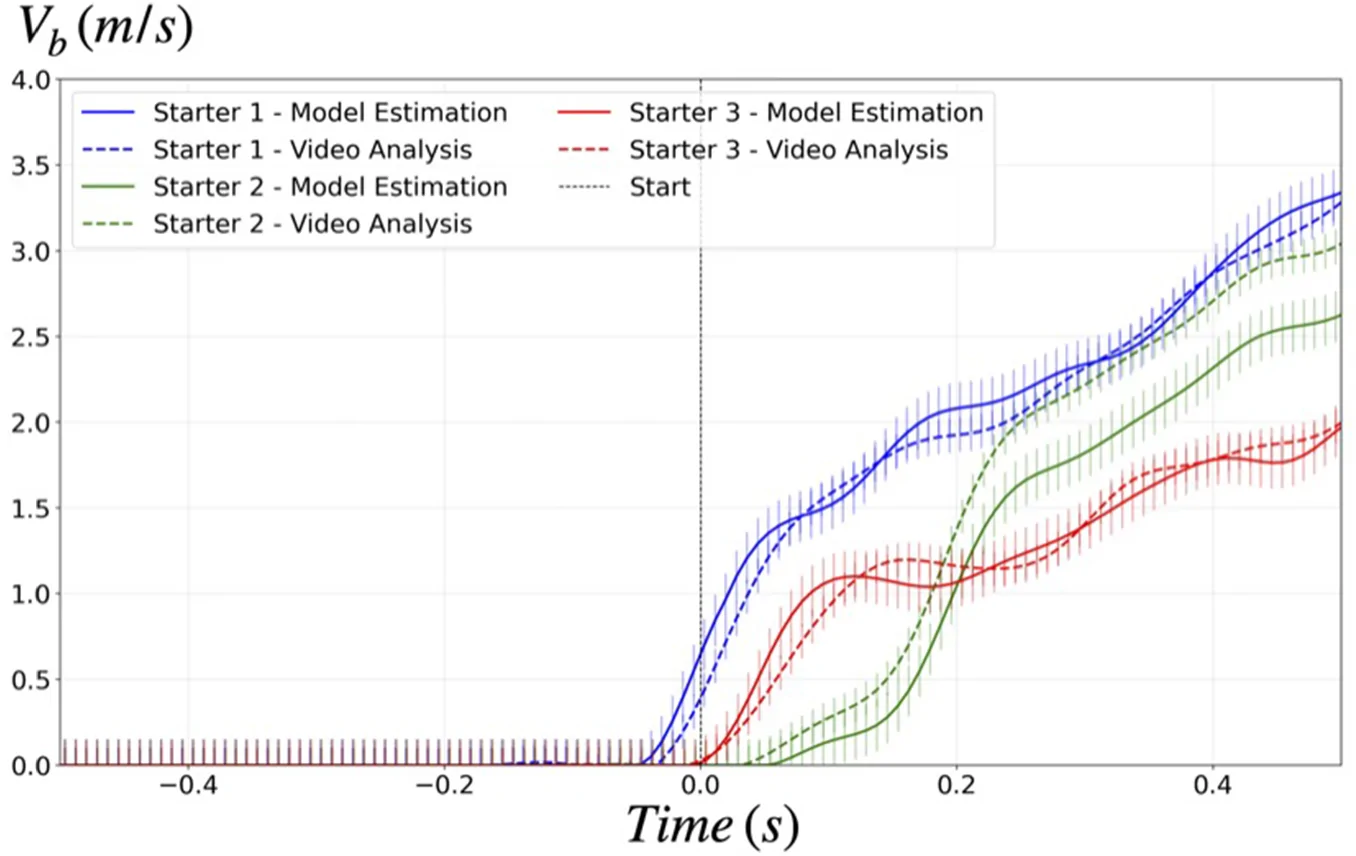
Comparison between model-predicted velocities (solid lines) and video-measured velocities (dashed lines) for the three starters. Vertical bars on the markers represent the propagated uncertainty from the video tracking and filtering procedure; the model bands, where visible, show the corresponding uncertainty propagated through equation (4.1).

### 4.3. The soft-impact limit: why legacy models set $V_{b}(t=0)=V_{0}$

As shown in figure 1c, several predictive models initialize the bicycle with a non-zero velocity at the official signal [6,7]. This choice, while ad hoc at first sight, can be rationalized by a soft-impact argument that captures the coupling between the rider and the bicycle at the instant of release.

Consider the rider (mass $M_{c}$) moving forward relative to the bicycle with velocity $V_{c}$ just before coupling, and the bicycle (mass $M_{b}$) momentarily at rest. Over a brief interval Δt—set by body and bicycle compliance and shorter than the time scale of steady pedalling—the rider ‘locks’ to the bicycle. Neglecting drivetrain work during Δt and treating the gate's external impulse as small over this short window, the longitudinal impulse–momentum balance for the coupled system gives, to leading order,

(4.2) $$(M_{c}+M_{b})\;V_{b}(0^{+})\;\approx \;M_{c}\;V_{c}(0^{-}),$$

so that the immediate post-coupling bicycle speed satisfies

(4.3) $$V_{b}(0^{+})\;\approx \;\frac{M_{c}}{M_{c}+M_{b}}\;V_{c}(0^{-}).$$

Equation (4.3) is the *soft-impact limit*: a fraction of the rider's pre-coupling forward velocity is transferred to the bicycle, yielding a lawful non-zero $V_{b}$ at the instant of motion onset.

A more refined statement includes the small external impulse from the gate, $J_{b}=\int_{0^{-}}^{0^{+}}F_{b}\;dt$, and any nascent crank impulse $J_{p}$ during Δt:

(4.4) $$(M_{c}+M_{b})\;V_{b}(0^{+})\;=\;M_{c}\;V_{c}(0^{-})\;-\;J_{b}\;+\;J_{p},$$

which is the integral version of equation (3.1). When $J_{b}$ and $J_{p}$ are negligible over the short coupling window, the soft-impact expression (equation (4.3)) is recovered. In our full framework, this limit corresponds to the early part of the transitional regime in which the inertial term $F_{i}$ is largest and the gate force is rapidly decaying. Thus, legacy models that prescribe $V_{b}(t=0)=V_{0}$ can be viewed as adopting the soft-impact outcome as an initial condition for the subsequent constant-acceleration phase, whereas our approach resolves that outcome dynamically from the measured forces. The soft-impact relation predicts only an instantaneous velocity if the pre-coupling CoM velocity is supplied, whereas equation (4.1) predicts the whole velocity trace from independently measured gate, CoM and crank inputs. In the validation window of figure 10, the full model reproduces both the onset and the subsequent growth of $V_{b}(t)$, while the soft-impact limit provides only a useful order-of-magnitude check on $V_{b}(t=0^{+})$. Finally, and more importantly, the full model allows a discussion on the optimization of the synchronization of the initial motion with the starting gate opening.

## 5. Optimization

We now use the validated model to derive practical, lawful gains during the first instants of the start. By varying initial position (offset), initiation timing and force-application profiles, we identify strategies that maximize early acceleration while preserving stability and compliance with regulations. The discussion emphasizes actionable implications for Starter 1, with a focus on velocity at the signal and the time to the 30 cm benchmark.

### 5.1. Start timing

Unlike a 100 m sprint in athletics, the track-cycling start is not a pure reflex task: a regular countdown and synchronized visual–auditory cues during the final 5 seconds allow anticipation. Performance therefore depends critically on how precisely initiation is phased with gate release, rather than on reaction time alone. Moreover, the bicycle is not invariably positioned exactly on the line at t=0. Small, lawful offsets enable momentum build-up provided the front wheel does not cross the line prematurely; loading the gate can assist this process but, if poorly controlled, the braking force may destabilize posture.

Figure 11 illustrates how advancing or delaying initiation modifies the total driving force relative to the gate's time-varying resistance. Only the timing shifts that satisfy the no-crossing condition at t=0 and do not require rearward motion after gate release are used in the optimization; larger shifts are retained only to illustrate sensitivity.

**Figure 11.**
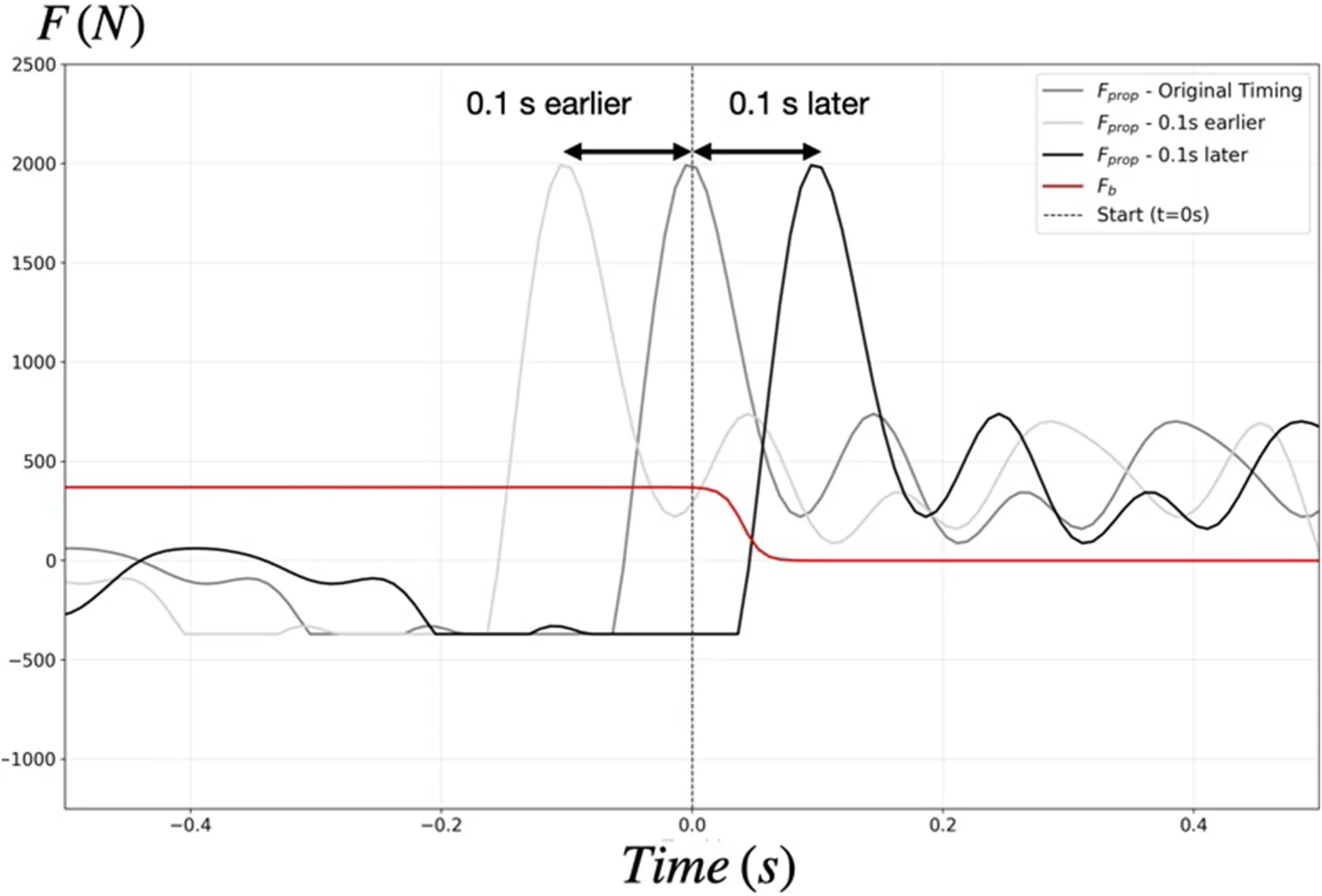
Total driving force for different initiation timings relative to the gate's braking capacity. Earlier or later onsets alter the overlap with the release window and hence the net drive at t≈0; timings outside the physically admissible window are used only as sensitivity cases.

#### 5.1.1. The zero offset limit

In the original performance the bicycle was set 6 cm behind the line, allowing some legal pre-start velocity. Here, we examine the limiting case in which the wheel is placed on the line (offset ≈0.1 cm). In this configuration, any pre-start motion would provoke a false start. Using the model, we scanned timing shifts around Starter 1's original pattern and identified the onset that minimizes the time to 30 cm while remaining legal (figure 12). The optimum is delayed by approximately 0.03 s relative to the original timing; it yields $V_{b}(t=0)\approx 0$ m s${}^{-1}$ and a 30 cm time of 0.22 s. Any earlier onset would push the wheel over the line.

**Figure 12.**
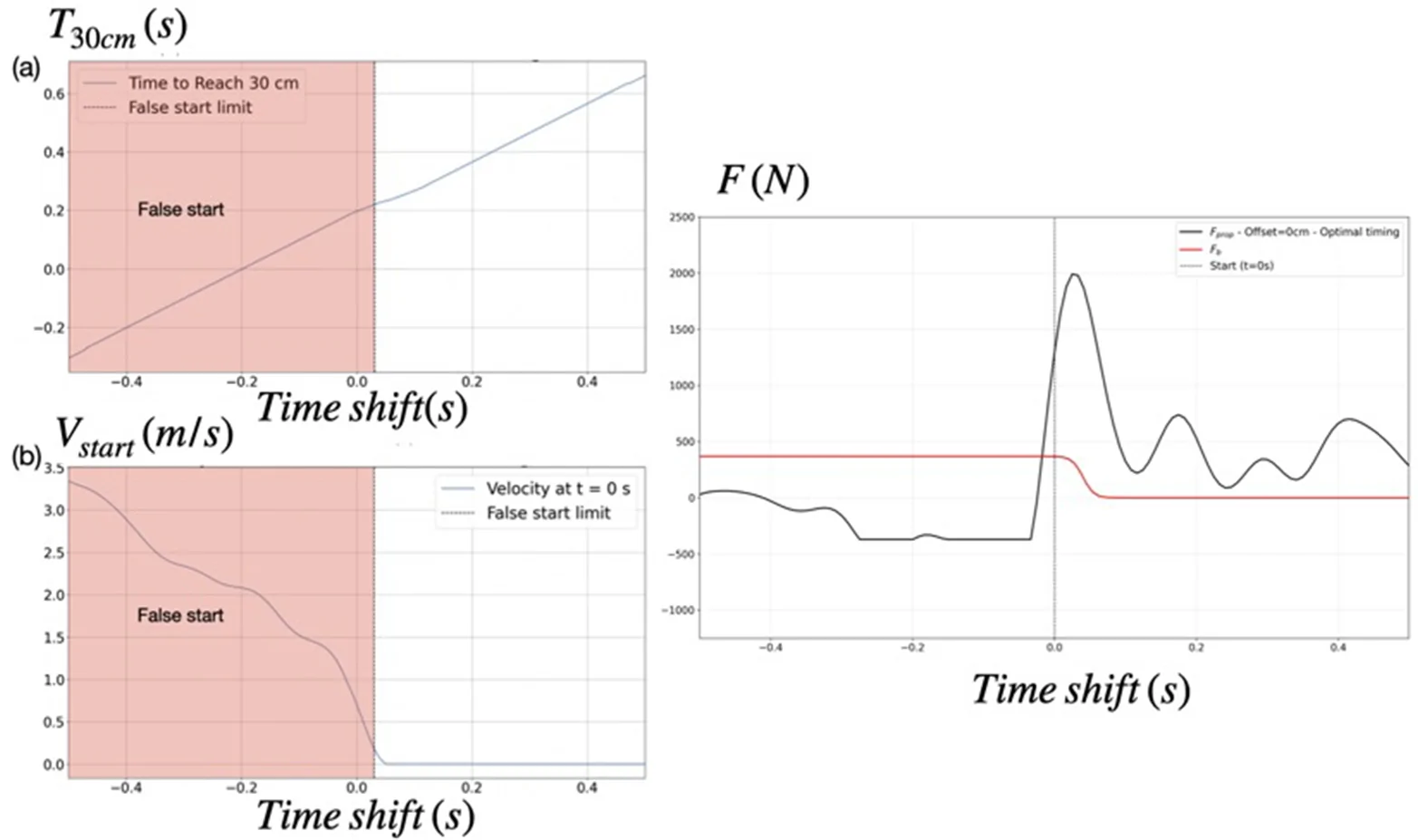
Optimization of initiation timing for zero offset. The 0 s shift corresponds to Starter 1's original timing. (a) Time to 30 cm versus timing shift. (b) Velocity at the signal versus timing shift. (c) Dynamics at the optimal timing that minimizes the 30 cm time while remaining legal.

This result also shows that, with a 6 cm offset, Starter 1 exploited only a fraction of the legally available distance to generate pre-start velocity. The model provides a direct way to tune onset to offset so as to balance legal acceleration against false-start constraints.

#### 5.1.2. Initial distance from the start line

Offsets are common in competition and depend on bicycle geometry and gate placement. Riders capable of loading the gate without destabilization can use a larger offset to initiate earlier and attain a non-zero $V_{b}(t=0)$; such behaviour is consistent with the British and Australian examples referenced in table 1. Figure 13 compares optimal timings for 0 and 10 cm offsets: an allowance of 10 cm enables initiation roughly 0.1 s earlier than at zero offset. Although table 1 lists offsets of up to 14 cm in our experiments, the offset range observed in competition is 0–10 cm. In this paragraph, we therefore restrict our analysis to this range, which is the relevant one for applications.

**Figure 13.**
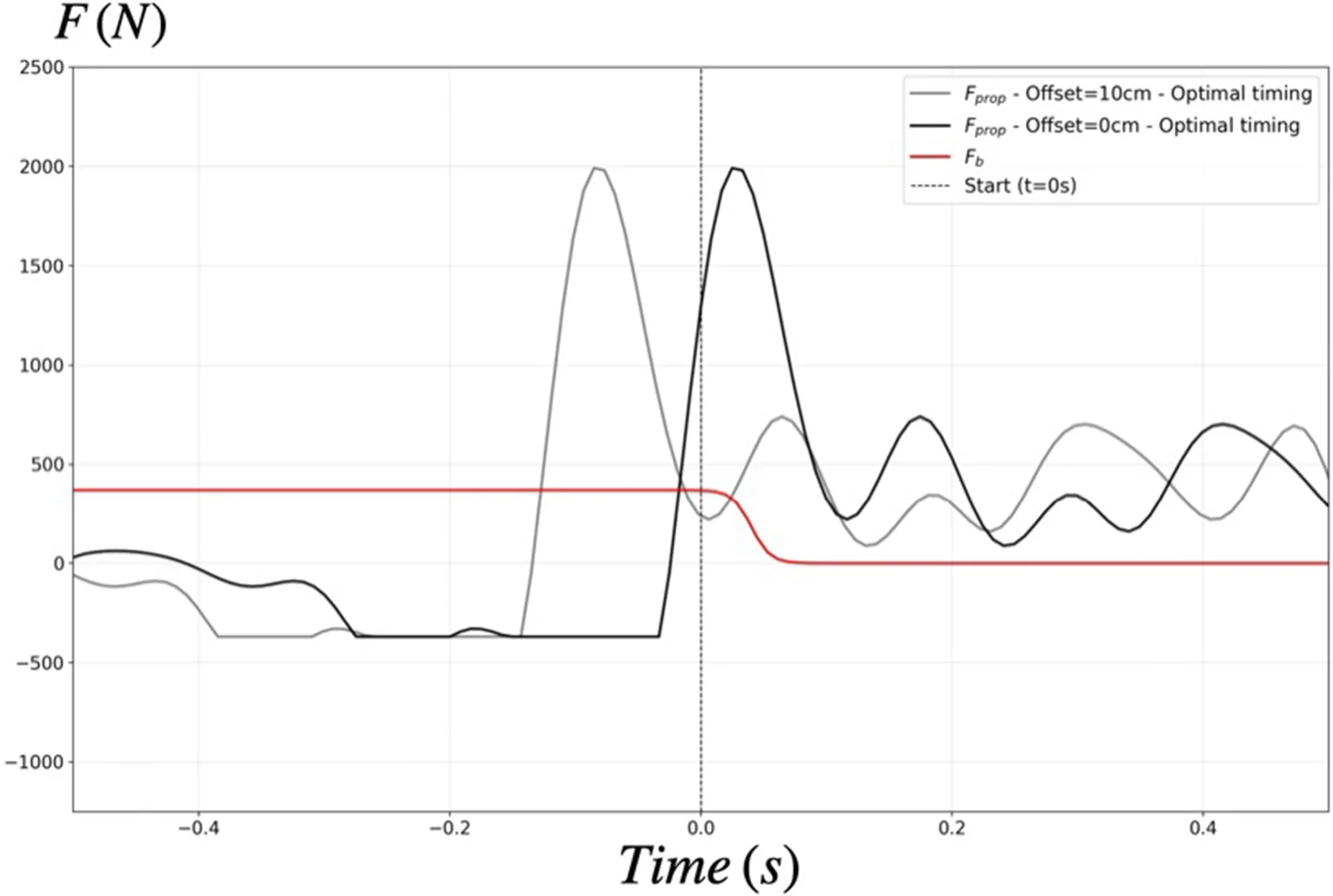
Comparison of optimal initiation timing for 0 cm and 10 cm offsets. A larger offset lawfully brings initiation forward and increases $V_{b}(t=0)$ within the admissible range.

The quantitative effect on $V_{b}(t=0)$ and the 30 cm time is summarized in table 2. Over the geometrically realistic range examined here, larger offsets permit higher legal start velocities and shorter 30 cm times. We have not interpreted this as a universal monotonic law: very large offsets would depend on the gate geometry, bicycle size and the rider's ability to load the gate without losing stability. In practice, the offset is not freely chosen by the rider, so the actionable variable remains the timing of the body motion for the offset imposed by the gate. The Australian benchmark in table 1 ($V_{start}=2.0$ m s${}^{-1}$ with a 4 cm offset) should therefore be read as an athlete-specific observation, not as a prediction from the Starter 1 parameters. Achieving such a value with a smaller offset would require a larger and more precisely phased CoM impulse than measured for Starter 1.

**Table 2.** Effect of initial distance on $V_{start}$ and the time $T_{30\;\text{cm}}$ to 30 cm (optimal admissible timing in each case).

initial distance (cm)	$V_{start}$ (m s${}^{-1}$)	$T_{30\;\text{cm}}$ (s)
0	0.0	0.22
6	1.3	0.18
10	1.5	0.16

### 5.2. Effect of other parameters on initial velocity

To assess other influences, we considered Starter 1 with a 6 cm offset and the previously optimal timing, and then perturbed each model parameter by +10% (table 3). The results isolate first-order sensitivities. The line ‘timing 0.1 s earlier’ is a sensitivity case, not a legal recommendation: at a 6 cm offset it would be very likely to move the wheel over the line before the signal unless the offset were larger or the onset were delayed.

**Table 3.** Sensitivity analysis (baseline: 6 cm offset with optimal timing). The 0.1 s earlier timing case is included to show sensitivity and would probably be a false start at this offset.

conditions	$V_{start}$ (m s${}^{-1}$)	variation rate (%)
model estimation	1.34	0.00
timing 0.1 s earlier	1.87	+39.55
${\ddot{X}}_{G}(t)+10\%$	1.48	+10.45
$\Gamma_{p}(t)+10\%$	1.36	+1.49
$M_{\text{cyclist}}+10\%$	1.36	+1.49
$M_{\text{bike}}+10\%$	1.33	−0.75
G+10%	1.32	−1.49
$F_{b,\max}+10\%$	1.31	−2.24
timing 0.1 s later	0.12	−91.04

The hierarchy is clear: enhancing CoM acceleration ${\ddot{X}}_{G}$ yields the largest legal gains among non-timing parameters; crank torque $\Gamma_{p}(t)$ and cyclist mass have modest first-order effects at fixed timing; bicycle mass and gear ratio exert only minor direct influence over this short window, although gearing interacts with torque and cadence beyond the present scope.

### 5.3. Optimization of standing-start performance

*Timing precision.* A shift of ±0.1 s about the optimum can change $V_{b}(t=0)$ by +39.55% or −91.04%. The legal performance envelope is therefore narrow, and the trade-off between early momentum and false-start risk is acute. Millisecond-level onset control, supported by real-time feedback, should be a training priority.

*Biomechanical levers.* The dominant non-timing lever is explosive CoM acceleration ${\ddot{X}}_{G}$, confirming the importance of coordinated whole-body motion during the transitional regime. By contrast, equipment choices that chiefly influence mass or static gearing contribute less to $V_{b}(t\;=\;0)$ over the first approximately 0.2 s.

*Regulatory constraints.* The gate's braking force sets a hard threshold for lawful motion onset. A moderate offset (e.g. 6 cm) can be exploited to produce substantial legal pre-start velocity, provided onset is synchronized with gate release and the wheel remains behind the line at t=0. Video-based feedback is recommended to align CoM dynamics and torque application with the release window.

*Practical implications.* Training should integrate (i) timing drills using auditory/visual cues with immediate feedback, and (ii) targeted strength and plyometric work to increase ${\ddot{X}}_{G}$. An integrated protocol that couples explosive movement training with timing feedback will align whole-body motion to the model-derived optimum and convert the available offset into lawful early gains.

*Effect of $V_{\text{start}}$ on total sprint time.* The present measurements focus on the first 30 cm, but a higher $V_{\text{start}}$ should not be dismissed as a local effect only. A simple estimate is obtained by assuming that the later speed–time curve is unchanged and that the advantage is carried into the first acceleration phase. For an early acceleration of approximately 5–8 m s${}^{-2}$, an extra 0.1–0.2 m s${}^{-1}$ at the signal corresponds to roughly 0.01–0.04 s saved in the launch phase. This is small in absolute terms but relevant in elite time trials, where final margins can be of the same order. A full-event prediction would require extending the present model to cadence, aerodynamic drag and pacing over the remainder of the race.

## 6. Conclusion

This study resolves the paradox of the track-cycling standing start—how a departure ‘from rest’ can present $V_{b}(t\;=\;0)\neq 0$—by making explicit the short transitional regime around the gate release preceding steady pedalling. Through direct characterization of the starting gate, high-speed kinematic analysis of elite specialist starters and instrumented measurements of crank torque, we separated and quantified the three concurrent contributions that govern the onset of motion: (i) braking by the gate ($F_{b}$), (ii) a body-induced inertial term ($F_{i}$) arising from rapid CoM motion relative to the bicycle and (iii) pedalling propulsion ($F_{p}$). The resulting mechanistic balance

$$M\;{\dot{V}}_{b}\;=\;F_{p}-F_{b}+F_{i},$$

captures the temporal ordering and relative magnitudes of these terms. In particular, we showed that $F_{i}$ dominates the first approximately 0.2 s, readily overcoming the brief, high plateau of $F_{b}$, while $F_{p}$ becomes primary as posture stabilizes and the system clears the gate. Model predictions agree closely with velocities obtained from videography, thereby providing a quantitative explanation of lawful non-zero $V_{b}(t\;=\;0)$ without resort to ad hoc initial conditions.

Building on this framework, we demonstrated how legal performance gains can be engineered by coordinating initiation timing with the gate's release window and by exploiting a finite offset behind the line. Optimization analyses revealed a narrow temporal margin: advances of order $10^{-2}$–$10^{-1}$ s can materially alter $V_{b}(t\;=\;0)$ and the time to 30 cm, but also approach the false-start boundary. Among non-timing variables, explosive CoM acceleration emerged as the most effective lever in the transitional regime, whereas gear ratio and mass exerted only minor first-order effects over this short interval. These findings offer clear practical guidance: training should prioritize millisecond-level timing control, video-based feedback to align whole-body motion with gate release, and targeted development of the neuromuscular qualities that amplify ${\ddot{X}}_{G}$; equipment choices should be made with an appreciation of their limited influence on the very earliest phase.

Beyond track cycling, the framework generalizes to other held or block-based starts in which an external constraint is released and a compliant rider–apparatus (or athlete–apparatus) coupling produces a soft-impact transfer of momentum. Athletics blocks, short-track speed skating, BMX gate starts and swimming block departures admit analogous decompositions into constraint, inertial and propulsive terms. Extending the present methodology to those contexts will require event-specific characterization of the ‘gate’ (e.g. block reaction in athletics, hinge dynamics in BMX) and early-phase propulsion (e.g. push-off kinetics on ice, block-to-flight mechanics in swimming), together with rule-bound timing windows. Such cross-disciplinary applications should clarify how coordination and timing—rather than power alone—govern start effectiveness and how lawful performance can be engineered under differing apparatus, surfaces and regulatory constraints.

## Figures and Tables

**Figure 14.**
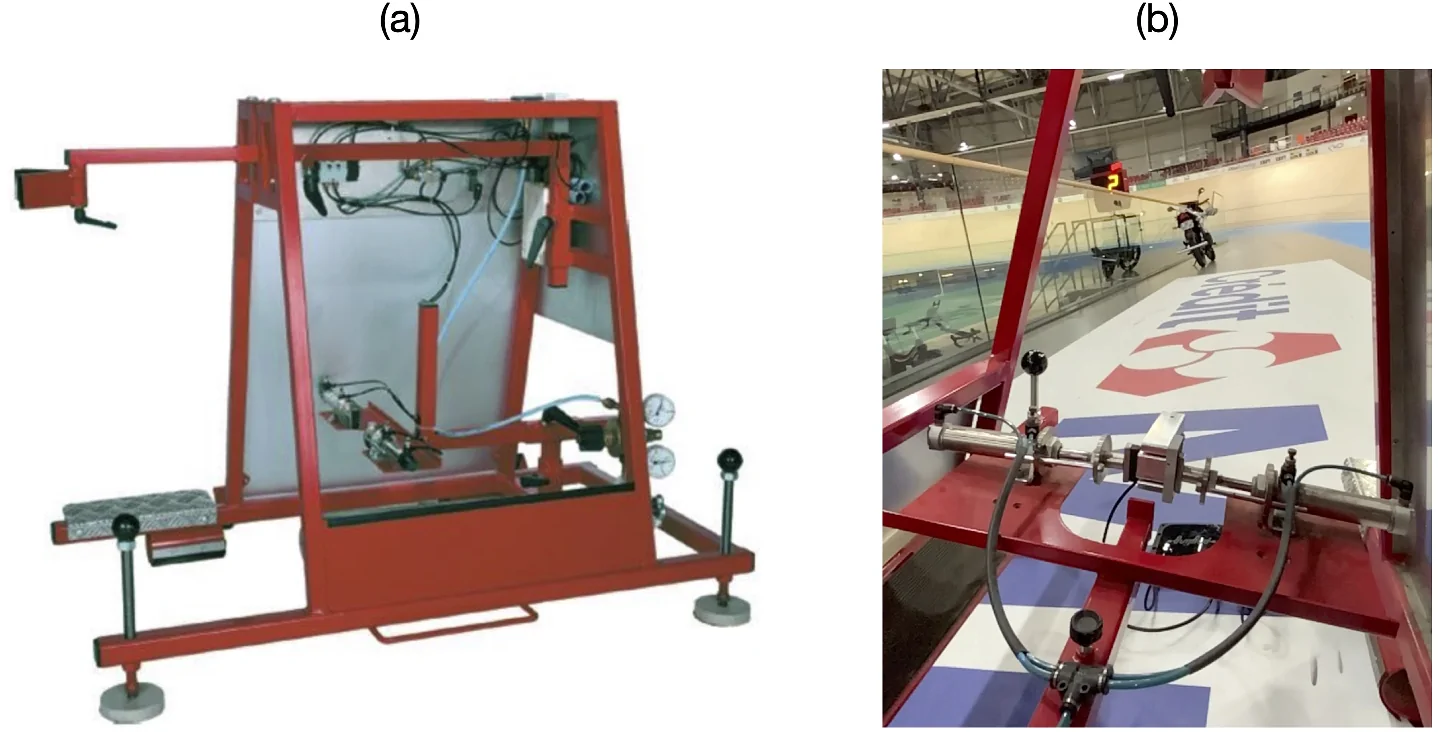
Swiss Timing starting gate. (a) Overall assembly. (b) Caliper jaws clamping the in-line force sensor used to measure the normal load as a function of regulator pressure.

**Figure 15.**
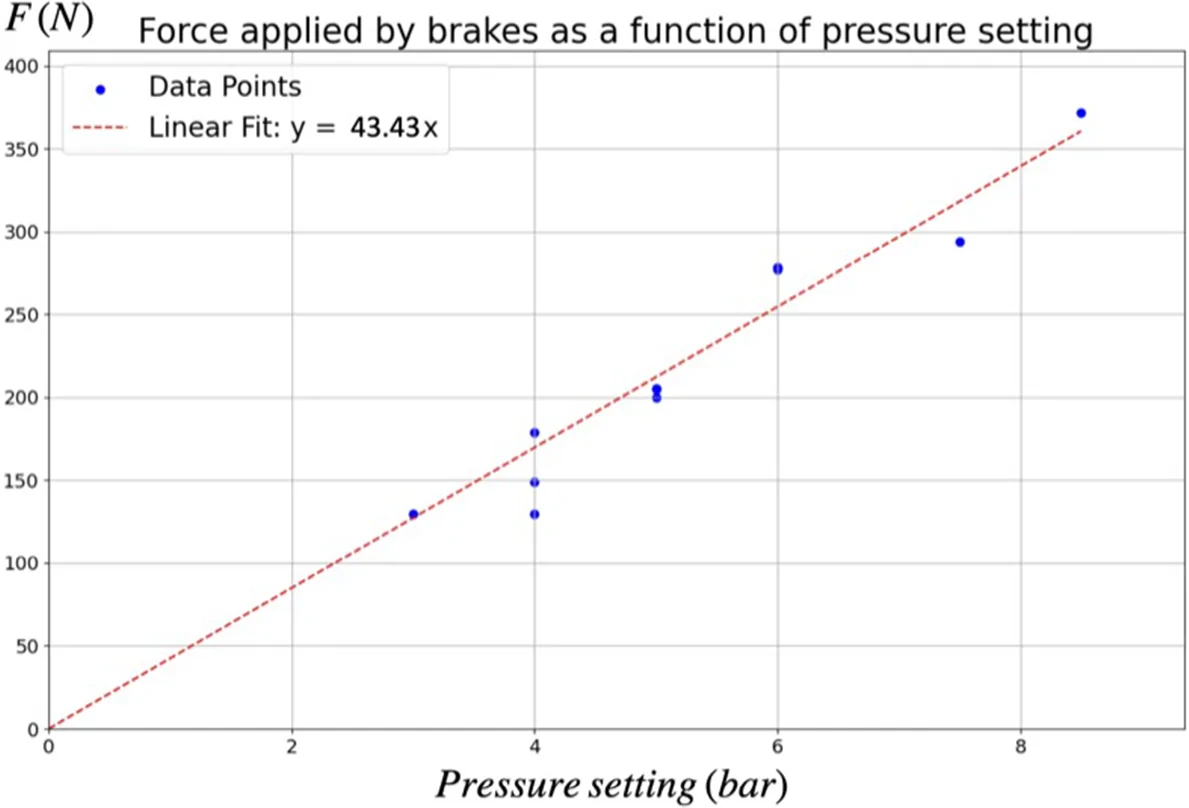
Normal force applied by the caliper as a function of regulator pressure (horizontal axis in bar). The relation is approximately linear over the tested range.

**Figure 16.**
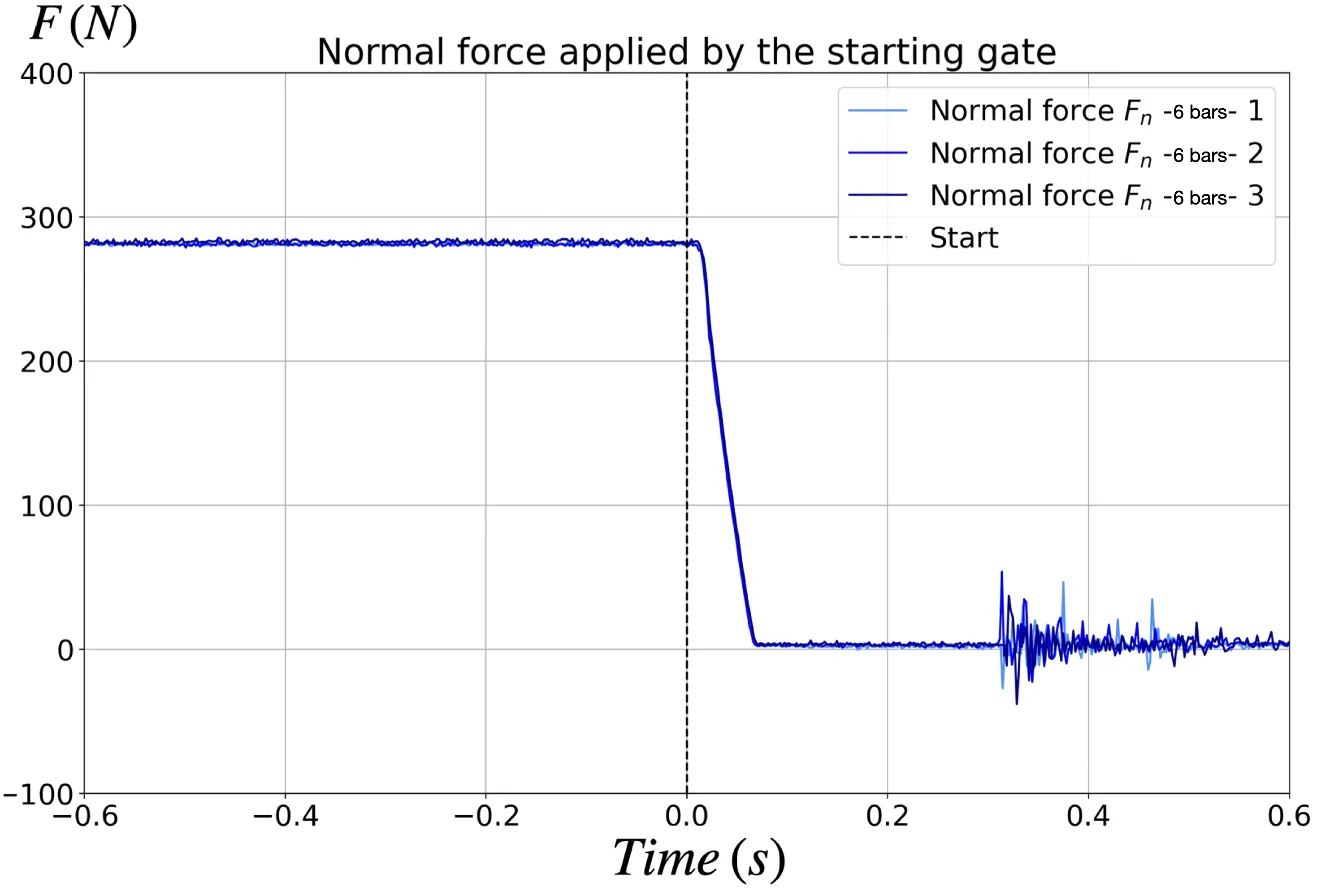
Normal force versus time for three repeats at 6 bar. The gate opens immediately after the signal and completes release in approximately 0.06 s.

## Data Availability

All data and codes are accessible with the following link: https://seminaris.polytechnique.fr/share/s/StBmFWWCSZaWRWq.

## Appendix A. Automatic starting gate

### A.1. Overview

In held starts, the rear wheel is secured by an automatic starting gate that releases at the official signal. The device used here was designed and developed by Swiss Timing in collaboration with the UCI. We characterize its mechanical action in order to parameterize the braking force $F_{b}$ employed in the main text.

### A.2. Methods

The Swiss Timing gate employs pneumatic actuators to clamp the rear wheel. Supply pressure was provided by a 200 bar nitrogen cylinder and regulated in the range of 4–8 bar (the standard competition setting is 6 bar).

Two measurements were undertaken (figure 14):

— *Normal force versus pressure.* A custom in-line force sensor (Phyling; 200 Hz) was placed between the caliper jaws to record the normal force during a start while the gate opened. Trials were performed at multiple regulator pressures. Synchronous high-speed video provided the timing of the acoustic/visual signal and the onset/cessation of clamping.
— *Limiting friction and effective coefficient.* A bicycle with an instrumented crankset was secured in the gate. An experimenter applied pedal force until the rear wheel slipped free. A simple transmission model (as in §3) mapped crank torque to the tangential force at the tyre–gate interface, yielding the limiting friction and the corresponding effective coefficient μ.

### A.3. Results

**Normal force and release timing.** The normal force increased approximately linearly with regulator pressure (figure 15); a least-squares fit yielded an affine dependence with slope ≈43.4 N bar${}^{-1}$. At 6 bar the measured normal load was $F_{n}\approx 278$ N. Time-resolved traces at 6 bar (figure 16) show that the gate begins to open immediately after the start signal and completes its release in ΔT≈0.06 s, after which the clamping force falls effectively to zero.

**Limiting friction and effective coefficient.** Slip-release tests at several pressures gave the limiting tangential force ($F_{tang}$) and the corresponding effective friction coefficient (table 4). The mean coefficient was μ=1.33, and the limiting friction increased with regulator pressure, as expected.

**Table A.1.** Slip-release measurements of the limiting tangential force and effective friction coefficient. The mean value μ=1.33 is used in the main-text model.

pressure setting (bar)	limiting $F_{tang}$(N)	$F_{n}$ (N)	effective coefficient μ
2.0	118.3	86.8	1.36
2.0	123.9	86.8	1.42
1.5	84.5	65.1	1.29
1.5	85.6	65.1	1.31
1.4	78.9	60.7	1.29
1.25	76.1	54.2	1.40
1.0	56.3	43.4	1.29
**mean**	—	—	**1.33**

### A.4. Interpretation and use in the model

The measurements support a simple Coulomb description of the braking action during clamping:

$$F_{b}=\mu \;F_{n},$$

with $F_{n}$ set by the regulator pressure and μ≈1.33. The time dependence of $F_{b}$ during opening is well represented by a smooth transition of duration ΔT≈0.06 s (see §3). These parameters—linear $F_{n}$–pressure scaling, mean μ and release duration—are those used in the dynamical model to reproduce the early force balance and to explain the brief, gate-limited window that precedes sustained pedalling.

## Appendix B. CoM reconstruction

Table 5 lists a summary of the relevant parameters for our study

**Table A.2.** Segment percentage of body mass and α (longitudinal CoM position from the proximal endpoint) from de Leva [11].

body segment	body mass (%)		longitudinal CoM position α (%)	
	male	female	male	female
calf	4.33	4.81	44.59	44.16
thigh	14.16	14.78	40.95	36.12
trunk	43.46	42.57	44.86	43.46
arm	2.71	2.55	57.72	57.54
forearm	1.62	1.38	45.74	45.59
head	6.94	6.18	/	/
hand	0.61	0.56	/	/
foot	1.37	1.29	/	/

Equations (B 1) and (B 2) define the computation used throughout:

(B 1)CoMi,X=Xproximal,i+αiXdistal,i−Xproximal,i,CoMi,Y=Yproximal,i+αiYdistal,i−Yproximal,i,(B 2)CoMX=∑imiCoMi,X∑imi,CoMY=∑imiCoMi,Y∑imi,

where $\alpha_{i}$ is the longitudinal position of the segment's CoM as a percentage of the segment's length from the proximal joint. We use standard anatomical terminology: for each limb segment, the *proximal* endpoint is the joint closer to the trunk (e.g. hip for the thigh, knee for the shank, shoulder for the upper arm, elbow for the forearm), and the *distal* endpoint is the joint farther from the trunk (e.g. knee for the thigh, ankle for the shank, elbow for the upper arm, wrist for the forearm).

### B.1. Signal processing

All position trajectories (front hub and digitized joints) were processed in the same way. First, outliers caused by temporary tracking loss were removed manually and replaced by local interpolation. Second, positions were filtered with a zero-phase Butterworth low-pass filter (second order, 12 Hz cut-off). Velocities were computed from the filtered positions using a second-order central finite difference

$$\dot{X}(t_{k})≃\frac{X(t_{k+1})-X(t_{k-1})}{2\Delta t},$$

with one-sided differences at the ends of each record. The resulting velocities were filtered with a zero-phase Butterworth low-pass filter (fourth order, 8 Hz cut-off). CoM accelerations used in $F_{i}=M_{c}{\ddot{X}}_{G}$ were obtained by applying the same central-difference procedure to the filtered CoM velocity. This processing was used for figures 3, 7 and 8.
